# Comment on “Adult onset of ganglioneuroblastoma of the adrenal gland: case report and review of the literature”

**DOI:** 10.1186/s40792-022-01502-w

**Published:** 2022-07-28

**Authors:** Christopher Stevens, Kevin Malone, Amro Saad Aldine, Octavio Arevalo

**Affiliations:** Louisiana State University Health Sciences Center-Shreveport, 501 Kings Highway, Shreveport, LA 71130 USA

**Keywords:** Ganglioneuroblastoma, Adrenal gland, Adult onset

## To the Editor

We have examined the case report and review article written by Bolzacchini et al**.** entitled “Adult onset of ganglioneuroblastoma of the adrenal gland: case report and review of the literature” [1]. The authors successfully presented a case of adrenal ganglioneuroblastoma in an adult patient, a finding that the authors claimed to have only been reported in the literature 16 times previously. However, in their literature review involving the previous cases of adult onset of adrenal ganglioneuroblastoma, we believe several grievous errors were made that unfortunately led to their conclusion being inaccurate and misleading.

In Table 3 of their article, the authors list their case and the supposedly 16 previous reported cases of adult-onset adrenal ganglioneuroblastoma, including the patient’s age (years) and sex of each case. However, in two of the cases they cited [2, 3], the tumor was identified as a ganglioneuroma in the patient, not a ganglioneuroblastoma, thus resulting in two cases in their review that should not have been included. In addition, the authors also mistakenly listed these two cases as occurring in males when both occurred in females [2, 3]. Their citation of a ganglioneuroblastoma case by Fujiwara et al. was also mistakenly listed as a male patient when the patient was female [4]. The first author’s name, year of the study, and age of the patient from reference 14 are also wrongly listed in Table 3. The study they cited, [5], was published by Koike et al. in 2003 and involved a 50-year-old male with ganglioneuroblastoma, but Bolzacchini et al. have the paper listed as being published by Kishikawa et al. in 1992, and the patient listed as a 29-year-old male. Another error was made when the authors recorded the patient’s age from reference 11, Cemron et al., as 58 years old; the patient was actually 54 years of age, according to the article the authors cited (6). In addition, in the paragraph preceding Table 3, the authors say that 14/17 of the cases listed in the table occurred in males, but the table shows that 13/17 cases occurred in males.

These mistakes resulted in a conclusion made by the authors that was inaccurate; this is especially seen in the relationship between patient sex and the presence of an adrenal ganglioneuroblastoma of adult onset. Additionally, the paper misidentified the number of times this tumor type had been reported in the literature at the time of 2015, the year of this paper’s publication. Due to the extreme rarity of cases for this type of tumor, this publication significantly impacted the statistics regarding features seen in ganglioneuroblastoma.

## Data Availability

Not applicable.
